# Quality indicator survey of clinical practice guidelines for esophagogastric junction cancer 2023

**DOI:** 10.1093/dote/doaf071

**Published:** 2025-09-08

**Authors:** Satoru Matsuda, Bas Wijnhoven, Florian Lordick, Pradeep Bhandari, Fenglin Liu, Ken Kato, Takuji Gotoda, Lorenzo Ferri, Hiroya Takeuchi, Yoshihiro Kakeji, Han-Kwang Yang, Yuko Kitagawa

**Affiliations:** Department of Surgery, Keio University School of Medicine, Tokyo, Japan; Department of Surgery, Erasmus MC Cancer Institute, Erasmus University Medical Center, Rotterdam, The Netherlands; Department of Oncology and University Cancer Center, Leipzig Leipzig University Medical Center, Comprehensive Cancer Center Central, Leipzig-Jena, Germany; Gastroenterology, Portsmouth University Hospital NHS Trust, Hampshire, UK; Second Department of Gastric Surgery, Fudan University Shanghai Cancer Center, Shanghai, China; Department of Oncology, Shanghai Medical College, Fudan University, Shanghai, China; Department of Gastrointestinal Medical Oncology, National Cancer Center Hospital, Tokyo, Japan; Department of Head and Neck, Esophageal Medical Oncology, National Cancer Center Hospital, Tokyo, Japan; Department of Gastroenterology, Cancer Institute Hospital Japanese Foundation for Cancer Research, Tokyo, Japan; Division of Thoracic and Upper Gastrointestinal Surgery, Montreal General Hospital, McGill University Health Centre, Montreal, Canada; Department of Surgery, Hamamatsu University School of Medicine, Hamamatsu, Japan; Division of Gastrointestinal Surgery, Department of Surgery, Kobe University Graduate School of Medicine, Kobe, Japan; National Cancer Center, Seoul, South Korea; Department of Surgery, Keio University School of Medicine, Tokyo, Japan

**Keywords:** endoscopy, esophagogastric junction cancer, guidelines, medical oncology, surgery

## Abstract

Clinical practice guidelines for esophagogastric junction cancer (EGJ GLs) were published in 2023. In order to evaluate how EGJ GLs have been adopted into clinical practice worldwide and to identify any outstanding clinical questions to be addressed in the next edition, this survey was conducted.

An electronic questionnaire was developed. The questionnaire comprised 16 questions designed to assess the adoption of the guideline. Responses were collected online. The survey was conducted by the EGJ working group of International Gastric Cancer Association (IGCA) following approval from the guideline committee of The International Society for Diseases of the Esophagus (ISDE).

As results, we received 344 valid and complete responses. 55% of respondents were from East Asia followed by Europe, Central/South America, and Central/West Asia. 80% of respondents recognized and followed the guidelines to some extent. There was still diversity in the extent of lymphadenectomy for EGJ cancers with an esophageal invasion of 2–4 cm. Although white light imaging (WLE) alone was recommended in the EGJ GLs, both WLE and image enhanced endoscopy were used in 86% of respondents. The perioperative treatment was shown to be highly diverse worldwide. While 50% of respondents provided perioperative chemotherapy, preoperative chemotherapy without adjuvant treatment and upfront surgery were still the first treatment option in 15% of respondents.

In conclusion, the current survey conducted by IGCA and ISDE identified the current standard and remaining issues of EGJ cancers.

## INTRODUCTION

Advances in multimodal therapies have helped improve the outcomes of patients with cancer of the esophagogastric junction (EGJ).^1^^,^^2^ However, current treatment strategies have become increasingly diverse and differ between geographical regions. Strategies for EGJ cancer vary by country and institution.^3^ Therefore, it will be crucial for oncologists to adopt a global, comprehensive approach to its treatment. To this end, the Upper Gastrointestinal (GI) Oncology Summit was organized as an international consensus meeting on EGJ cancer at the 2023 International Gastric Cancer Conference with the aim to establish international clinical practice guidelines.

In the conference, expert panel members were selected and proposed the clinical questions in the field of EGJ cancer including endoscopy, medical oncology, and surgery. Based on the systematic review, they proposed the recommendation statements. Finally, as a result of a Delphi process, clinical practice guidelines for EGJ cancer were published in 2023 and endorsed by the International Gastric Cancer Association (IGCA) and International Society for Diseases of the Esophagus (ISDE).^4^

Since it was the first international clinical practice guldens in the upper GI field, it is unclear whether these guidelines are recognized and used in daily clinical practice. Furthermore, it is not clear to what extent they are being followed. In order to evaluate how EGJ Guidelines (GLs) have been adopted into clinical practice worldwide and to identify any outstanding clinical questions to be addressed in the next edition, the working group was developed by IGCA and quality indicator survey was planned to be conducted.

## METHODS

After the first EGJ cancer guidelines were published, an EGJ working group was established within the IGCA. The working group includes two endoscopists (TK and PB), two medical oncologists (FL and KK), and three surgeons (BW, FL, and SM), who were the main contributors to the first guidelines. During the development of the questionnaires, these seven members reviewed them to ensure their appropriateness in evaluating the clinical adaptation of the current guidelines and addressing the remaining issues for the second edition.

The questionnaires were uploaded to the software platform SurveyMonkey. The link was distributed via the IGCA, ISDE, Japanese Gastric Cancer Association, and Japan Esophageal Society membership email lists. After the deadline, members of the EGJ working group reviewed and analyzed the answers. The questionnaire comprised 16 questions designed to assess the adoption of the guideline. The questions asked are as listed in the Supplementary Table S1.

Since the survey did not involve patient data or samples, approval from an institutional review board was waived.

## RESULTS

**Fig. 1:**
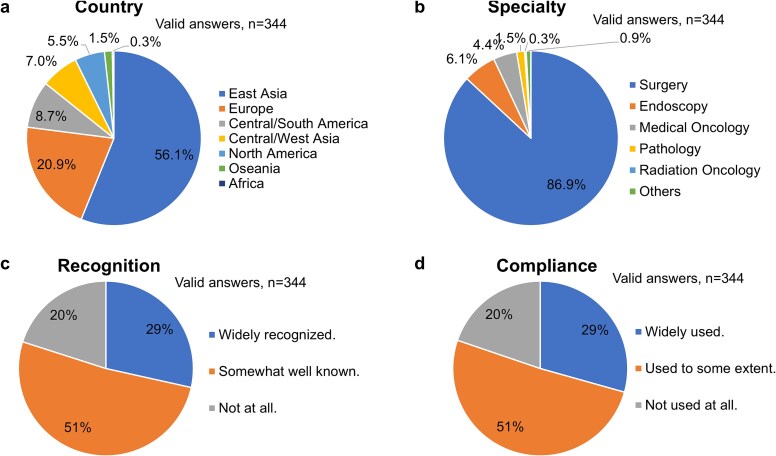
Background of respondens and recognition of guidelines.

We received 344 valid and complete responses. An answer was considered valid if one or more questions were answered. All answers were recorded on 258 sheets. A total of 55% of respondents were from East Asia followed by Europe, Central/South America, and Central/West Asia (Fig. 1a). Of respondents from East Asia, Japan (n = 139) and China (n = 49) were the major contributors. The countries where there were more than 10 responses were Italy, Germany, Brazil, Turkey, and United States. When specialty was asked, the surgeons exceeded 86%, followed by endoscopists, and medical oncologists (Fig. 1b).

A total of 80% of respondents recognized and followed the guidelines to some extent (Fig. 1c and d). Reasons for not following the guideline included the presence of the regional or National guidelines that they need to follow, lack of awareness, and the weak recommendations for most of the recommendations.

**Fig. 2:**
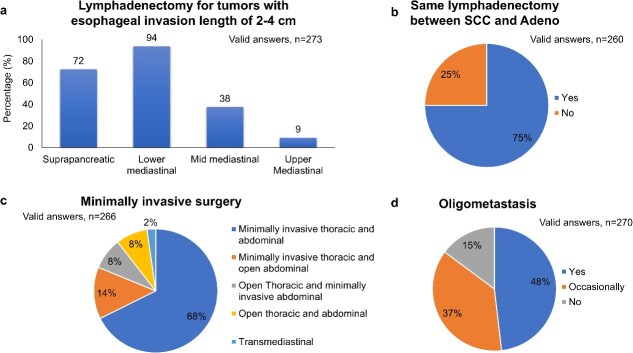
Results of survey: Surgery.

Surgery-related clinical questions are described in Figure 2. There was still diversity in the extent of lymphadenectomy for EGJ cancers with an esophageal invasion of 2–4 cm. Consistent to the recommendation in the guideline, the same lymphadenectomy was performed for squamous cell carcinoma and adenocarcinoma located at EGJ in 75% (Fig. 2b). The implementation of minimally invasive surgery for EGJ cancer is shown in Fig. 2c. Some 92% of respondents underwent total or hybrid minimally invasive approaches. Finally, the majority of respondents (85%) considered surgery for oligometastasis of EGJ cancer (Fig. 2d). The geographical difference between East Asia and non-East Asia was shown in Table 1. The distribution of answers was almost identical between groups.

**Table 1:** Answer from East Asia and Non-East Asia

		East Asia N = 193	Non-East Asia N = 151
Surgery			
Which lymph node station are you dissecting in patients with EGJ cancers showing an esophageal invasion length of 2–4 cm? (Multiple answers were accepted.)	Suprapancreatic	102 (70%)	95 (74%)
	Lower mediastinal	135 (93%)	120 (94%)
	Mid mediastinal	38 (26%)	64 (50%)
	Upper Mediastinal	18 (12%)	7 (5%)
Are you dissecting the same lymph node region of EGJ squamous cell carcinoma and EGJ adenocarcinoma?	Yes	94 (69%)	101 (82%)
	No	43 (31%)	22 (22%)
Which surgical approach is preferred for EGJ cancer? (Hand assisted approached is grouped with minimally invasive approach.)	Minimally invasive thoracic and abdominal	104 (73%)	76 (62%)
	Minimally invasive thoracic and open abdominal	20 (14%)	16 (13%)
	Open Thoracic and minimally invasive abdominal	7 (4%)	16 (13%)
	Open thoracic and abdominal	6 (5%)	15 (12%)
	Transmediastinal	6 (4%)	0 (0%)
Do you consider local treatment for gastroesophageal junction cancer with oligo metastasis?	Yes	75 (54%)	55 (42%)
	Occasionally	45 (32%)	55 (42%)
	No	20 (14%)	20 (16%)
Endoscopy			
Which method are you using for the detection of superficial neoplasia (cancer/high grade dysplasia) at the EGJ?	WLE alone	11 (10%)	17 (20%)
	WLE and IEE	101 (90%)	70 (80%)
Which method are you using to determine the extent of superficial neoplasia (cancer/high grade dysplasia) at the EGJ?	WLE alone	9 (8%)	15 (18%)
	WLE and IEE	97 (92%)	69 (80%)
	Others	0 (0%)	2 (2%)
Which criterion are you using for curative resection of neoplasia at the EGJ?	A) An intramucosal carcinoma of any size.	15 (15%)	10 (9%)
	B) Carcinoma with a submucosal invasion depth of <500 um; and the tumor diameter < 3 cm; negative resection margins; with no evidence of lymphovascular invasion or poorly differentiated components.	43 (43%)	61 (57%)
	Either A) or B)	42 (42%)	35 (32%)
	Other criteria	0 (%)	2 (2%)
What % of lesions are removed by Piecemeal (EMR) vs en-bloc (ESD)? (%)	0–20	4 (5%)	47 (59%)
	21–40	2 (3%)	5 (6%)
	41–60	5 (6%)	8 (10%)
	61–80	2 (3%)	7 (9%)
	81–100	64 (83%)	13 (16%)
Medical oncology			
Are you using the same chemotherapy regimens as those established for patients with unresectable advanced or recurrent gastric adenocarcinoma in patients with esophagogastric junction adenocarcinoma and esophageal adenocarcinoma?	Yes	102 (86%)	78 (70%)
	No	16 (14%)	34 (30%)
Which perioperative treatment for resectable, locally advanced esophagogastric junction cancer?	Preoperative chemotherapy	20 (16%)	17 (15%)
	Preoperative chemoradiotherapy	2 (1%)	13 (11%)
	Preoperative chemotherapy + adjuvant chemotherapy	56 (45%)	63 (55%)
	Preoperative chemoradiotherapy + adjuvant chemotherapy	15 (12%)	17 (15%)
	Upfront surgery + adjuvant chemotherapy	31 (25%)	5 (4%)
	Surgery alone	1 (1%)	0 (0%)
Which biomarkers are you testing before first-line systemic therapy for unresectable esophagogastric junction cancer? (Multiple answers were accepted.)	HER2	123 (100%)	114 (99%)
	PD-L1 CPS	118 (96%)	82 (71%)
	dMMR/MSI	106 (86%)	98 (85%)
	Claudin 18.2	94 (76%)	36 (31%)

EGJ, esophagogastric junction; IEE, image enhanced endoscopy; WLE, white light imaging; EMR, endoscopic musosal resection; ESD, endoscopic submucosal resection.

**Fig. 3:**
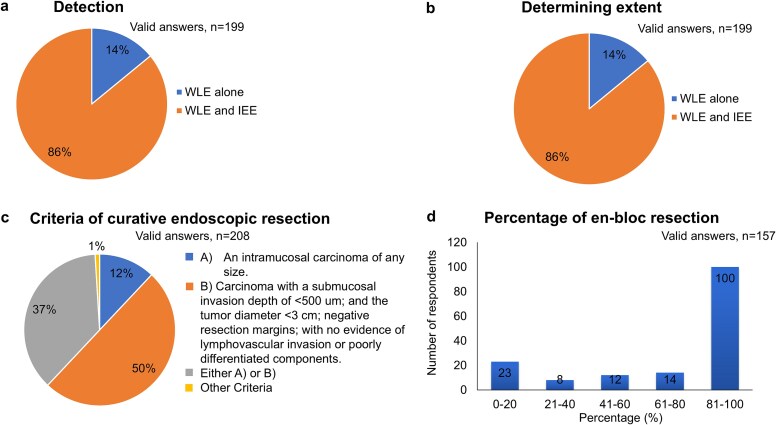
Results of survey: Endoscopy.

Although white light imaging (WLE) alone was recommended in the guideline, both WLE and image enhanced endoscopy (IEE) were used in 86% of respondents (Fig. 3a). Consistent to the recommendation, 86% of clinicians were using both WLE and IEE to determine the extent of neoplasia (Fig. 3b). Regarding the criteria of an endoscopic curative resection, only 37% of respondents were following the recommendation of the guideline (Fig. 3d). On the other hand, there was a considerable difference in the percentage of en bloc resection in endoscopy (Table 1).

**Fig. 4:**
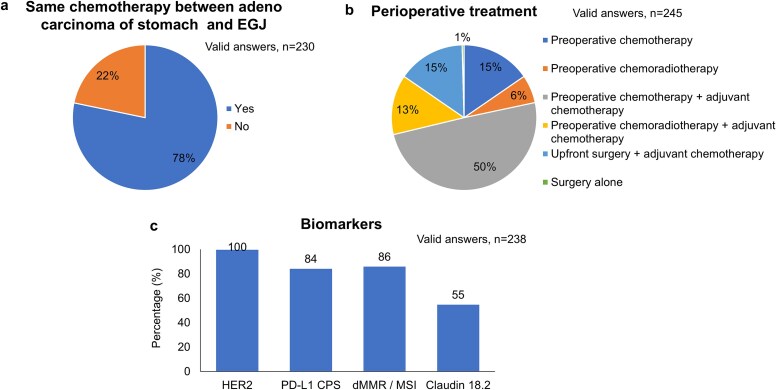
Results of survey: Medical oncology.

Around 78% of respondents were providing the same chemotherapy regimen to patients with EGJ adenocarcinoma as for gastric cancer (Fig. 4a). The perioperative treatment was shown to be highly diverse worldwide. While 50% of respondents provided perioperative chemotherapy, preoperative chemotherapy without adjuvant treatment, and upfront surgery were still the first treatment option in 15% of respondents (Fig. 4b). Biomarker-testing was done for HER-2, PD-L1, mismatch repair, and Claudin 18–2 (Fig. 4c). When, the differences between East Asia and the non-East Asian regions were reviewed, the evaluation of Claudin 18.2 testing was relatively lower in non-East Asia (Table 1).

## DISCUSSION

The QI survey of the first clinical practice guidelines for esophageal and gastric junction (EGJ) cancer was successfully conducted, receiving a sufficient number of responses from around the world. Although nearly half of the respondents were from East Asia, responses were also received from Europe, North America, South America, and other regions. The results also showed that surgeons were the main contributors to EGJ cancer clinical practice. However, based on the result that 80% of respondents recognized and used the current clinical practice guidelines, we concluded that they are familiar with them and that the guidelines are adopted in clinical practice to some extent in various countries.

In the questions for surgery, the responses were fitting to the recommendations in all of four clinical questions (CQs). Regarding the range of LN dissection, although the lower mediastinal LN dissection was conducted in 94% of respondents, the suprapancreatic LN dissection was done in 72% indicating that the field of lymphadenectomy still widely varies for EGJ cancer with the esophageal invasion length of 2–4 cm. An international observational cohort study to determine the distribution of LN metastases in patients with resectable esophageal or EGJ carcinoma will provide further guidance.^5^ The surgical approach was one of the debatable CQ in the previous Upper GI Oncology Summit 2023. As of 2023, several randomized phase III studies have shown that the minimally invasive thoracic or abdominal approach improved short-term outcomes.^6^^,^^7^ Therefore, it was weakly recommended to conduct thoracoscopic (robotic) esophagectomy in patients with resectable EGJ cancer. In 2024, JCOG1409 was published, showing that thoracoscopic esophagectomy was non-inferior to open esophagectomy with regard to overall survival.^8^ Furthermore, in a randomized control trial, robotic esophagectomy lead to more successful left recurrent laryngeal nerve lymph node dissection than thoracoscopic esophagectomy.^9^ In fact, in this questionnaire, 82% of respondents preferred minimally invasive in thoracic approach reflecting the increase in the adaptation of thoracoscopic or robotic surgery worldwide.

WLE was weakly recommended, but the survey showed that clinicians were using both WLE and IEE for the detection of early neoplasia. Recently, most scopes have been equipped with IEE, which allows endoscopists to use it routinely. However, clinicians need to carefully evaluate whether IEE improves the accuracy of detecting neoplasms at the EGJ. The question regarding the criterion for curative resection of neoplasia at the EGJ was another one which was not in line with the recommendation. Only 37% of respondents chose the answer in which either intramucosal carcinoma or tumours with submucosal invasion with specific criteria were considered curative resection. This was mainly due to the lack of reliable data on the indications and criteria for curative endoscopic resection of malignant neoplasms arising in the EGJ. It is expected that criteria for EGJ cancer will be established in the future. In terms of the treatment, the 100 out of 157 respondents are adopting en-bloc resection in 80% or higher percentage of patients in clinical practice. On the other hand, there was a considerable difference in the percentage of en-bloc resection in endoscopy. In the near future, the treatment procedure will be standardized along with the indication and diagnostic criteria.

As weakly recommended in the guidelines, clinicians are accepting to use same chemotherapy regimens as those established for gastric cancer. On the other hand, efficacy of chemotherapy is not always similar across subgroups including location of the tumor (EGJ versus gastric), indicating that the recommendation statement might need to be updated.^10^^,^^11^ The perioperative treatment was highly diverse, while half of respondents were adopting the perioperative chemotherapy. Since the voting was conducted in 2023, a land mark study has proved to improve the survival using the triplet chemotherapy with compared to the neoadjuvant chemoradiotherapy.^2^ As the survival advantage of perioperative chemotherapy comparing to upfront surgery plus adjuvant therapy has been underway, which will further facilitate the standardization of perioperative treatment for EGJ cancer.^12^ As strongly recommended, the biomarkers were assessed in most of all markers while there was the room for improvement for Claudin 18 testing especially in non-East Asian countries.

Several limitations and remaining issues need to be acknowledged. Total number of respondents was 344 which is likely to be a small proportion of the member societies. Also, the denominator and characteristics of responders and non-responders could not be evaluated. Most of the respondents could be experts who were interested or involved in the development of the guideline in 2023. To evaluate the more details for each society, the next survey could be distributed through regional associations of upper GI cancer. When the reasons why the EGJ GLs were not used in their practice, the respondents pointed out the lack of awareness, and the necessity to follow the regional guidelines not the international guidelines. As a future perspective, it would be valuable to review the domestic GLs and clarify the similarity and difference among GLs, which will help to identify the role of international clinical practice guidelines for EGJ cancer. The classification of EGJ still diverse. Either Siewert or Nishi classification is used in various society, and the third classification ‘gastro oesophageal junction zone, GOJZ’ has been developed based on the Kyoto consensus.^13^^,^^14^ In order to uniform the classification, the further biological assessments would be needed.

In conclusions, the current survey conducted by IGCA and ISDE identified the current standard and remaining issues of EGJ cancers. The thorough review of regional guidelines and revisiting the discussion about the classification of EGJ cancer would facilitate to develop more standardized treatment for EGJ based on the EGL GLs.

## Additional information

This survey was edited by the Esophagogastric Junction Caner Working Group of International Gastric Cancer Association and supported by the Guideline Working Group of The International Society for Diseases of the Esophagus.

## Supplementary Material

Supplementary_Table_cleaned_doaf071

## Notes

Financial support: None declared.

Conflicts of interest: Dr Lordick reports personal fees from Amgen, personal fees from Astellas, personal fees and scientific grants from Astra Zeneca, personal fees from Art Tempi, personal fees and grants from Beigene, personal fees and scientific grants from BMS, personal fees from Boehringer Ingelheim, personal fees and scientific grants from Daiichi Sankyo, personal fees from Eli Lilly, personal fees from Elsevier, personal fees and grants from Gilead personal fees from Iomedico, personal fees from Incyte, personal fees from MedUpdate, personal fees from Merck Serono, personal fees from MSD, personal fees from PAGE, personal fees from Roche, personal fees from Servier, personal fees from Springer Nature, personal fees from StreamedUp!, personal fees from Servier, outside the submitted work. Dr Kato reports lecture fees and research funding from BMS, consulting fees and research funding from MSD, consulting fees and research funding from BeiGene, consulting fees from Roche, consulting fees and research funding from AstraZeneca, consulting fees and research funding from Bayer, lecture fees and research funding from Ono, lecture fees from Taihol, research funding from Chugai, research funding from Shionogi, outside the submitted work. Dr Kitagawa reports Grants and lecture fee from Asahikasei, Grants and lecture fee from Taiho, Grants and lecture fee from Chugai, Grants and lecture fee from Kaken, Grants and lecture fee from Kowa Company, Grants from Otsuka, Grants from Kyouwa Hakkou Kirin, Grants from Sumitomo, Grants from Terumo Corporation, lecture fee from Ethicon, lecture fee from Ono, lecture fee from Otsuka Pharmaceutical Factory, lecture fee from Olympus, lecture fee from Cardinal Health, lecture fee from Shionogi, lecture fee from BMS, lecture fee from MSD, lecture fee from Aska, lecture fee from Miyarisan, lecture fee from Toray, lecture fee from Daiichi Sankyo, lecture fee from Chugai Foundation for Innovative Drug Discovery Science, lecture fee from NipponKayaku, lecture fee from EA pharma, lecture fee from intuitive Surgical, lecture fee from Takeda, lecture fee and research funding from Sysmex corporation, lecture fee from Tsumura, lecture fee from AI medical service, lecture fee from Eisai, research funding from Medicaroid Corporation, outside the submitted work.
