# Supplementary material for: Quality indicator survey of clinical practice guidelines for esophagogastric junction cancer 2023
Source: Dis Esophagus. 2025 Sep 8;38(5):doaf071. doi: 10.1093/dote/doaf071 (PMC12490043; doi:10.1093/dote/doaf071)
Supplement: Supplementary_Table_cleaned_doaf071 [file supplementary_table_cleaned_doaf071.docx]

**Supplementary Table 1.** List of questionnaires

| General | |
| --- | --- |
| 1 | What country do you work in? |
| 2 | Please select your specialty. |
| 3 | Is EGJ Cancer Clinical Practice Guidelines 2023 recognized in your institution (region/country)? |
| 4 | Is EGJ Cancer Clinical Practice Guidelines 2023 used in practice at your institution (region/country)? |
| 5 | (For those who chose “Not used at all.” in Q4) Please share why you didn't use the guidelines. |
| Surgery | |
| 6 | Which lymph node station are you dissecting in patients with EGJ cancers showing an esophageal invasion length of 2-4 cm? (You may select multiple answers.) |
| 7 | Are you dissecting the same lymph node region of EGJ squamous cell carcinoma and EGJ adenocarcinoma? |
| 8 | Which surgical approach is preferred for EGJ cancer? (Hand assisted approached is grouped with minimally invasive approach.) |
| 9 | Do you consider local treatment for gastroesophageal junction cancer with oligo metastasis? |
| Endoscopy | |
| 10 | Which method are you using for the detection of superficial neoplasia (cancer/ high grade dysplasia) at the GEJ ? |
| 11 | Which method are you using to determine the extent of superficial neoplasia　(cancer/ high grade dysplasia) at the GEJ? |
| 12 | Which criterion are you using for curative resection of neoplasia at the GEJ? |
| 13 | What % of lesions are removed by Piecemeal (EMR) vs en-bloc (ESD) |
| Medical oncology | |
| 14 | Are you using the same chemotherapy regimens as those established for patients with unresectable advanced or recurrent gastric adenocarcinoma in patients with esophagogastric junction adenocarcinoma and esophageal adenocarcinoma? |
| 15 | Which perioperative treatment for resectable, locally advanced esophagogastric junction cancer? |
| 16 | Which biomarkers are you testing before first-line systemic therapy for unresectable esophagogastric junction case? (You may select multiple answers.) |
